# Overexpression of ZEB2 in Peritumoral Liver Tissue Correlates with Favorable Survival after Curative Resection of Hepatocellular Carcinoma

**DOI:** 10.1371/journal.pone.0032838

**Published:** 2012-02-29

**Authors:** Mu-Yan Cai, Rong-Zhen Luo, Jie-Wei Chen, Xiao-Qing Pei, Jia-Bin Lu, Jing-Hui Hou, Jing-Ping Yun

**Affiliations:** 1 State Key Laboratory of Oncology in South China, Guangzhou, China; 2 Department of Pathology, Cancer Center, Sun Yat-Sen University, Guangzhou, China; 3 Department of Ultrasound, Cancer Center, Sun Yat-Sen University, Guangzhou, China; Northwestern University, United States of America

## Abstract

**Background:**

ZEB2 has been suggested to mediate EMT and disease aggressiveness in several types of human cancers. However, the expression patterns of ZEB2 in hepatocellular carcinoma (HCC) and its effect on prognosis of HCC patients treated with hepatectomy are unclear.

**Methodology/Principal Findings:**

In this study, the methods of tissue microarray and immunohistochemistry (IHC) were utilized to investigate ZEB2 expression in HCC and peritumoral liver tissue (PLT). Receiver operating characteristic (ROC), spearman's rank correlation, Kaplan-Meier plots and Cox proportional hazards regression model were used to analyze the data. Up-regulated expression of cytoplasmic/nuclear ZEB2 protein was observed in the majority of PLTs, when compared to HCCs. Further analysis showed that overexpression of cytoplasmic ZEB2 in HCCs was inversely correlated with AFP level, tumor size and differentiation (*P*<0.05). Also, overexpression of cytoplasmic ZEB2 in PLTs correlated with lower AFP level (*P*<0.05). In univariate survival analysis, a significant association between overexpression of cytoplasmic ZEB2 by HCCs/PLTs and longer patients' survival was found (*P*<0.05). Importantly, cytoplasmic ZEB2 expression in PLTs was evaluated as an independent prognostic factor in multivariate analysis (*P*<0.05). Consequently, a new clinicopathologic prognostic model with cytoplasmic ZEB2 expression (including HCCs and PLTs) was constructed. The model could significantly stratify risk (low, intermediate and high) for overall survival (*P* = 0.002).

**Conclusions/Significance:**

Our findings provide a basis for the concept that cytoplasmic ZEB2 expressed by PLTs can predict the postoperative survival of patients with HCC. The combined cytoplasmic ZEB2 prognostic model may become a useful tool for identifying patients with different clinical outcomes.

## Introduction

Hepatocellular carcinoma (HCC) is one of the most prevalent cancer types, and both the incidence and mortality rates of HCC have been steadily increasing in recent years [1]. Unfortunately, the long-term survival of patient with HCC remains unsatisfactory despite recent advances in surgical techniques and medical treatment [2]. This poor outcome is primarily related to the high incidence of recurrence and metastasis [3], [4]. Recurrence and/or metastasis of HCC are mainly intrahepatic, which suggests that the peritumoral liver tissue may be a favorable soil for the spreading tumor cells [5]. Biomarkers that could define the recurrence and metastatic potential of HCC may develop appropriate therapeutic regimens in the earlier course of this cancer; however, promising predictors that can be widely used in clinical settings are substantially limited [6], [7], [8].

Zinc finger E-box binding homeobox 2 (ZEB2, also known as SIP1) is a member of ZEB family of transcriptional factors. ZEB2 protein contains a central homeodomain, CtBP-binding and Smad-interacting domains and two zinc finger clusters each at either end [9], [10]. ZEB2 directly binds proximal E-boxes within the E-cadherin gene (*cdh1*) promoter and mediates transcriptional repression by recruiting corepressor complexes [11]. ZEB2 was originally identified as a binding partner of R-Smads, and shown to be part of the TGF-β pathway frequently involved in tumorigenesis [10]. hTERT repression in breast cancer cells could be partly mediated by ZEB2 in a TGF-β dependent manner [12]. In clonal HCC cells, ZEB2 was also found to be a mediator of hTERT repression by analysis of senescence arrest [13]. The relevance of ZEB2 protein to tumor progression has been studied in several types of human cancers. High ZEB2/E-cadherin ratio correlated with the aggressive phenotype and poor patient survival in breast and ovarian carcinomas [14]. Elevated ZEB2 transcripts were examined in renal cell carcinomas in a hypoxia-inducible factor 1 alpha-dependent manner [15]. Sayan et al indicated that overexpression of ZEB2 was an independent prognostic factor in bladder cancer and positively correlated with poor therapeutic outcome [16]. In addition, immunohistochemical analysis of oral squamous cell carcinomas revealed that ZEB2 protein was detected in a relatively low proportion of tumors and its expression correlated with poor prognosis [17]. Up to the present, however, the protein expression state of ZEB2 in HCC and the clinicopathologic/prognostic significance of this state have not been explored.

In the current study, immunohistochemistry (IHC) and tissue microarray were utilized to examine the distribution and frequency of ZEB2 expression in our HCC and peritumoral liver tissue (PLT) cohort. In order to avoid predetermined cutpoint, receiver operating characteristic (ROC) curve analysis was applied to define the cutoff score for separating ZEB2 overexpressed tumors from ZEB2 normally expressed tumors. Subsequently, the clinicopathologic/prognostic significance of ZEB2 expression in HCCs and PLTs was investigated.

## Methods

### Ethics statement

The study was approved by the Institute Research Medical Ethics Committee of Sun Yat-Sen University. No informed consent (written or verbal) was obtained for use of retrospective tissue samples from the patients within this study, most of whom were deceased, since this was not deemed necessary by the Ethics Committee, who waived the need for consent. All samples were anonymised.

### Patients and tissue specimens

In our study, the paraffin-embedded pathologic specimens from 248 patients with HCC were obtained from the archives of Department of Pathology, Sun Yat-Sen University Cancer Center, Guangzhou, China, between 1997 and 2008. The cases selected were based on distinctive pathologic diagnosis of HCC, undergoing primary and curative resection for tumor without preoperative anticancer treatment, availability of resection tissue and follow-up data. These HCC cases included 220 (88.7%) men and 28 (11.3%) women, with mean age of 47.8 years. Average follow-up time was 31.8 months (median, 26.0 months; range, 1.0 to 86.0 months).

Patients whose cause of death remained unknown or patients with neoadjuvant and adjuvant therapy were excluded from our study. Clinicopathologic characteristics for these patients including age, sex, hepatitis history, alpha-fetoprotein (AFP), liver cirrhosis, tumor number, size, differentiation, stage, vascular invasion and relapse were collected and detailed in Table 1. Tumor differentiation was based on the criteria proposed by Edmonson and Steiner [18]. Tumor stage was defined according to American Joint Committee on Cancer/International Union Against Cancer tumor-node-metastasis (TNM) classification system [19]. In addition, for ZEB2 western blotting analysis, fresh tissue specimens from 12 patients with HCC who underwent surgical resection were collected in 2011. Institute Research Medical Ethics Committee of Sun Yat-Sen University Cancer Center granted approval for this study.

**Table 1:** Correlation of ZEB2 expression with clinicopathologic variables.

		Cytoplasmic overexpression of ZEB2 (%)				Nuclear overexpression of ZEB2 (%)			
Variables		TT	*P* *	PLT	*P* *	TT	*P* *	PLT	*P* *
Age (years)			0.677		0.707		0.650		0.935
≤47.8†	123	76 (61.8)		60 (48.8)		8 (6.5)		28 (22.8)	
>47.8	125	74 (52.9)		58 (46.4)		10 (8.0)		29 (23.2)	
Sex			0.106		0.182		0.980		0.089
Male	220	137 (62.3)		108 (49.1)		16 (7.3)		47 (21.4) (49.1)	
Female	28	13 (46.4)		10 (35.7)		2 (7.1)		10 (35.7)	
HbsAg			0.362		0.769		0.221		0.873
Positive	216	133 (61.6)		102 (47.2)		14 (6.5)		50 (23.1)	
Negative	32	17 (53.1)		16 (50.0)		4 (12.5)		7 (21.9)	
AFP (ng/ml)			0.001		0.033		0.099		0.160
≤20	119	85 (71.4)		65 (54.6)		12 (10.1)		32 (26.9)	
>20	129	65 (50.4)		53 (41.1)		6 (4.7)		25 (19.4)	
Liver cirrhosis			0.216		0.740		0.582		0.701
Yes	179	104 (58.1)		84 (46.9)		14 (7.8)		40 (22.3)	
No	69	46 (66.7)		34 (49.3)		4 (5.8)		17 (24.6)	
Tumor size (cm)			0.049		0.943		0.249		0.180
≤5	133	88 (66.2)		63 (47.4)		12 (9.0)		35 (26.3)	
>5	115	62 (53.9)		55 (47.8)		6 (5.2)		22 (19.1)	
Tumor multiplicity			0.543		0.722		0.161		0.802
Single	140	87 (62.1)		68 (48.6)		13 (9.3)		33 (23.6)	
Multiple	108	63 (58.3)		50 (46.3)		5 (4.6)		24 (22.2)	
Differentiation			0.000		0.880		0.530		0.220
Well-moderate	148	103 (69.6)		71 (48.0)		12 (8.1)		38 (25.7)	
Poor-undifferentiated	100	47 (47.0)		47 (47.0)		6 (6.0)		19 (19.0)	
Stage			0.072		0.641		0.442		0.563
I-II	173	111 (64.2)		84 (48.6)		14 (8.1)		38 (22.0)	
III -IV	75	39 (52.0)		34 (45.3)		4 (5.3)		19 (25.3)	
Vascular invasion			0.148		0.094		0.381		0.341
Absent	170	108 (63.5)		87 (51.2)		14 (8.2)		42 (24.7)	
Present	78	42 (53.8)		31 (39.7)		4 (5.1)		15 (19.2)	
Relapse			0.356		0.504		0.898		0.359
Absent	148	93 (62.8)		73 (49.3)		11 (7.4)		37 (25.0)	
Present	100	57 (57.0)		45 (45.0)		7 (7.0)		20 (20.0)	

*Chi-square test;

† Mean age; HbsAg, hepatitis B surface antigen; AFP, alpha-fetoprotein; TT, tumor tissue; PLT, peritumoral liver tissue.

### Western blotting analysis

Equal amounts of whole tissue lysates were resolved by SDS-polyacrylamide gel electrophoresis (PAGE) and electrotransferred on a polyvinylidene difluoride (PVDF) membrane (Pall Corp., Port Washington, NY). The tissues were then incubated with primary anti-ZEB2 (Cat. No. HPA003456, Sigma, St. Louis, MO, 1∶1000 dilution). The immunoreactive signals were detected with enhanced chemiluminescence kit (Amersham Biosciences, Uppsala, Sweden). The procedures followed were conducted in accordance with the manufacturer's instructions.

### Tissue microarray (TMA) construction

Tissue microarray was constructed as the method described in our previous study [20]. In brief, formalin-fixed, paraffin-embedded tissue blocks and the corresponding H&E-stained slides were overlaid for TMA sampling. The slides were reviewed by a senior pathologist (M-Y. C.) to determine and mark out representative tumor areas. Duplicates of 0.6 mm diameter cylinders were punched from representative tumor areas and from adjacent non-malignant liver tissue of individual donor tissue block and re-embedded into a recipient paraffin block at defined position, using a tissue arraying instrument (Beecher Instruments, Silver Spring, MD).

### Immunohistochemistry (IHC)

The TMA slides were dried overnight at 37°C,deparaffinized in xylene, rehydrated through graded alcohol, immersed in 3% hydrogen peroxide for 20 minutes to block endogenous peroxidase activity, and antigen-retrieved by pressure cooking for 3 minutes in ethylenediamine tetraacetic acid (EDTA) buffer (pH = 8.0). Then the slides were preincubated with 10% normal goat serum at room temperature for 30 minutes to reduce nonspecific reaction. Subsequently, the slides were incubated with rabbit polyclonal anti-ZEB2 (Cat. No. HPA003456, Sigma, St. Louis, MO, 1∶100 dilution) which could recognize only one band corresponding to ZEB2 molecular weight (i.e., 136 kDa), anti-E-cadherin (Dako, Glostrup, Denmark, 1∶50 dilution) and anti-Vimentin (Dako, Glostrup, Denmark, 1∶200 dilution) for 2 hours at room temperature, respectively. The slides were sequentially incubated with a secondary antibody (Envision; Dako, Glostrup, Denmark) for 1 hour at room temperature, and stained with DAB (3,3-diaminobenzidine). Finally, the sections were counterstained with Mayer's hematoxylin, dehydrated, and mounted. A negative control was obtained by replacing the primary antibody with normal rabbit or mouse IgG. Known immunostaining positive slides were used as positive control.

### IHC evaluation

Immunoreactivity for ZEB2 protein was evaluated in semi-quantitative method as described previously [21]. Each TMA spot was assigned an intensity score from 0–3 (I0, I1–3) and proportion of tumor cells for that intensity over the total number of tumor cells was recorded as 5% increments from a range of 0–100 (P0, P1–3). A final H score (range 0–300) was achieved by adding the sum of scores obtained for each intensity and proportion of area stained (H score = I1XP1+I2XP2+I3XP3).

### Selection of cutoff score

**Figure 1:**
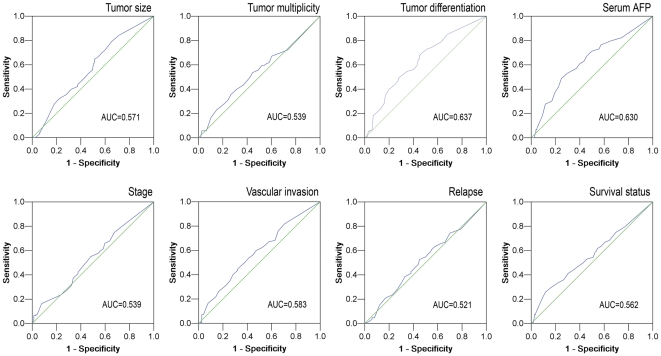
Receiver operating characteristic curves were created to determine the cutoff score for overexpression of cytoplasmic ZEB2 in HCCs. The sensitivity and specificity for each outcome were plotted and the areas under curve (AUCs) were indicated: tumor size (*P* = 0.053), tumor multiplicity (*P* = 0.286), tumor differentiation (*P*<0.0001), serum AFP (*P*<0.0001), stage (*P* = 0.330), vascular invasion (*P* = 0.036), relapse (*P* = 0.581) and survival status (*P* = 0.091).

ROC curve analysis was utilized to determine cutoff value for separating tumors with ZEB2 overexpression from tumors with ZEB2 normal expression by using the 0,1-criterion [22]. At the ZEB2 score, the sensitivity and specificity for each outcome under study was plotted, thus generating various ROC curves (Figure 1). The score was selected as the cutoff value, which was closest to the point with both maximum sensitivity and specificity. Tumors designated as “normal expression” for ZEB2 were those with scores below or equal to the cutoff value, while tumors with “overexpression” of ZEB2 were those with scores above the value. In order to perform ROC curve analysis, the clinicopathologic features were dichotomized: AFP level (≤20 ng/ml, or >20 ng/ml), tumor size (≤5 cm, or >5 cm), tumor multiplicity (single or multiple), tumor grade (well-moderately or poorly-undifferentiated), stage (I+II or III+IV), vascular invasion (absence or presence), relapse (absence or presence) and survival status (death due to HCC or censored).

### Statistical analysis

Statistical analyses were performed using the SPSS software program (SPSS Standard version 13.0, SPSS Inc, Chicago, IL). ROC curve analysis was applied to determine the cutoff value for ZEB2 overexpression by the 0,1-criterion, and the areas under curves (AUCs) were calculated. The correlations between ZEB2 expression and other variables were analyzed using Spearman rank test. The statistical significance of the correlation between ZEB2 expression and disease-specific survival was estimated by the log-rank test. Multiple Cox proportional hazards regression was carried out to identify the independent factors which had a significant impact on patient overall survival. A difference was considered significant if the *P* value from a two-tailed test was less than 0.05.

## Results

### The protein expression levels of ZEB2 in liver tissues by western blotting analysis

We examined the expression levels of ZEB2 protein in 12 additional pairs of HCC and matched adjacent liver tissues by Western blotting. The results showed that the ZEB2 antibody used in this study recognized only one band by Western blotting, and down-regulated expression of ZEB2 was detected in 9/12 (75.0%) cases of primary HCC tissues compared to adjacent non-neoplastic liver tissues (Figure S1).

### Expression patterns of ZEB2 in HCC and PLT by immunohistochemistry

**Figure 2:**
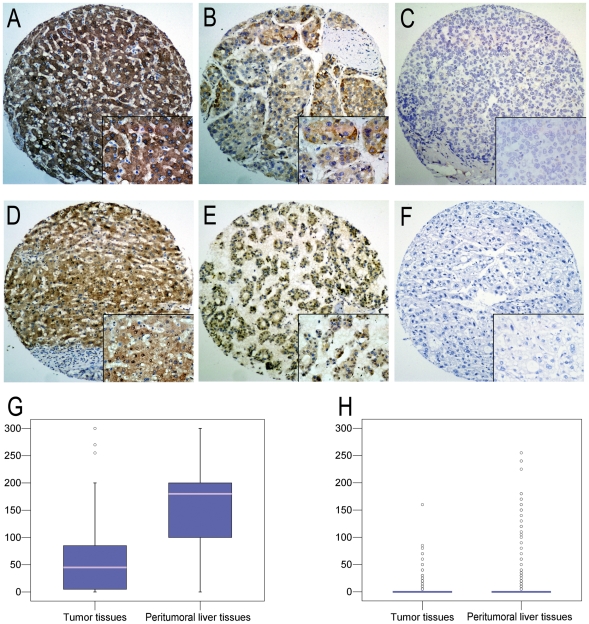
The expression patterns of ZEB2 in HCC and peritumoral liver tissues by IHC. (A) Overexpression of Cytoplasmic ZEB2 was shown in the peritumoral liver tissues (case 52), in which more than 95% hepetocytes revealed positive staining of ZEB2 in cytoplasm (×100). (B) The tumor tissues (case 37) demonstrated overexpression of cytoplasmic ZEB2, in which more than 85% of tumor cells showed immunoreactivity of ZEB2 in cytoplasm (×100). (C) Normal expression of ZEB2 was observed in a HCC case (case 52), in which less than 10% of tumor cells showed immunoreactivity of ZEB2 in cytoplasm (×100). (D) Nuclear overexpression of ZEB2 was shown in the peritumoral liver tissues (case 89), in which more than 80% hepetocytes revealed positive staining of ZEB2 in nuclei (×100). (E) The tumor tissues (case 76) demonstrated overexpression of nuclear ZEB2, in which more than 70% of tumor cells showed immunoreactivity of ZEB2 in nuclei (×100). (F) Negative expression of ZEB2 was observed in a HCC case (case 89, ×100). Representative sites with higher (inset, ×400) magnification were shown. (G) The box plot shows the mean staining intensity of cytoplasmic ZEB2 from HCC and peritumoral liver tissues (*P*<0.0001). (H) The box plot demonstrates the range of nuclear ZEB2 expression within each group (HCC, *n* = 248; peritumoral liver tissue, *n* = 248; *P*<0.0001).

ZEB2 staining was mainly on the cytoplasm (Figure 2A–2C) and/or nuclei (Figure 2D–2F) of tumor cells or hepatocytes. Most of the stromal cells were negative staining, though sporadic positive staining on these cells was also observed. For cytoplasmic expression, semi-quantitation of HCC and PLT in our cohort demonstrated a mean ZEB2 staining intensity of 53.4% [SE (standard error), 3.42%] and 151.1% (SE, 5.15%), respectively (Wilcoxon exact test, *P*<0.001, Figure 2G). For nuclear expression, the mean staining intensity of ZEB2 in HCCs was 5.0% (SE, 1.04%), which was significantly lower than those in PLTs (mean, 17.5%; SE, 3.00%; Wilcoxon exact test, *P*<0.001, Figure 2H). Further analysis showed that cytoplasmic expression levels of ZEB2 in peritumoral liver tissues positively correlated with the levels in the nuclei (r = 0.206, *P* = 0.001) and that there was no significant correlation between cytoplasmic and nuclear expression of ZEB2 in tumor tissues (r = 0.060, *P* = 0.303).

### Selection of cutoff scores for ZEB2 overexpression

To identify a single, optimal cutoff value for overexpression, ROC curve analysis was employed to determine the cutoff score for expression of ZEB2 in various patterns. The ROC curves for each clinicopathologic characteristics clearly show the point on the curve closest to (0.0, 1.0) which maximizes both sensitivity and specificity for the outcome as described in our previous study [23]. Tumors with scores above the obtained cutoff value were considered as ZEB2 overexpression leading to the greatest number of tumors classified based on clinical outcome presence or absence. According to ROC curve analysis, H score for cytoplasmic ZEB2 expression in HCC tissues above the cutoff value 70 was defined as overexpression and the corresponding AUCs were described in Figure 1. Similarly, tumors designated overexpression for cytoplasmic ZEB2 in PLT, nuclear ZEB2 in HCC, and nuclear ZEB2 in PLT were those with scores above the value of 165, 5 and 10, respectively (data not shown).

### Correlation between ZEB2 expression and HCC patients' clinicopathologic characteristics and survival

**Figure 3:**
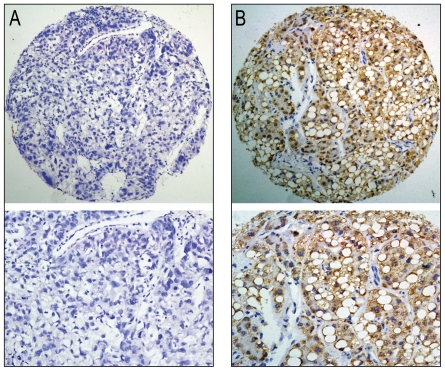
The cytoplasmic overexpression of ZEB2 in HCC tissues inversely correlated with tumor differentiation. (A) Negative expression of cytoplasmic ZEB2 was demonstrated in a poorly-differentiated HCC case (case 68, ×100). (B) Overexpression of cytoplasmic ZEB2 was observed in a well-differentiated HCC case (case 53), in which more than 95% of tumor cells showed immunoreactivity of ZEB2 protein in cytoplasm (×100). Representative sites in HCC tissue with higher (×200) magnification were shown.

**Figure 4:**
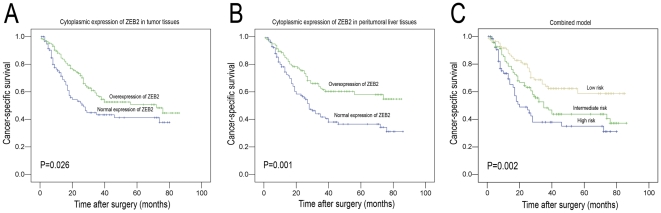
Kaplan-Meier survival analysis according to cytoplasmic ZEB2 expression in 248 patients with hepatocellular carcinoma (log-rank test). (A) *Cytoplasmic expression of ZEB2 in tumor tissues*, probability of survival of all patients with HCC: normal expression of ZEB2, *n* = 98; overexpression of ZEB2, *n* = 150 (*P* = 0.026). (B) *Cytoplasmic expression of ZEB2 in peritumoral liver tissues*, probability of survival of all patients with HCC: normal expression of ZEB2, *n* = 130; overexpression of ZEB2, *n* = 118 (*P* = 0.001). (C) Comparison of overall survival according to a combined cytoplasmic ZEB2 prognostic model (including tumorous and peritumoral expression): low risk, n = 86; intermediate risk, n = 95; high risk, n = 67 (*P* = 0.002).

In HCC tissues, further correlation analysis revealed that overexpression of cytoplasmic ZEB2 was significantly associated with serum AFP levels, tumor size and differentiation (*P*<0.05, Table 1, Figure 3). Furthermore, Kaplan–Meier analysis established that the mean disease-specific survival time for patients with HCC who overexpressed ZEB2 in cytoplasm was 53.8 months, compared to 41.8 months for patients with HCC who normally expressed ZEB2 in cytoplasm (*P* = 0.026, log-rank test, Table 2, Figure 4A).

**Table 2:** Univariate analysis of ZEB2 expression and clinicopathologic variables in 248 patients with primary hepatocellular carcinoma (log-rank test).

Variables	All cases	Mean survival (months)	Median survival (months)	*P* value
Age (years)				0.329
≤47.8*	123	52.8	72.0	
>47.8	125	47.1	35.0	
Sex				0.589
Male	220	50.6	NR	
Female	28	49.7	40.0	
HbsAg				0.743
Positive	216	50.4	40.0	
Negative	32	45.6	30.0	
AFP (ng/ml)				0.000
≤20	119	64.7	NR	
>20	129	36.6	25.0	
Liver cirrhosis				0.836
Yes	179	48.9	38	
No	69	50.5	40	
Tumor size (cm)				0.000
≤5	133	58.9	76.0	
>5	115	40.3	24.0	
Tumor multiplicity				0.000
Single	140	60.4	NR	
Multiple	108	37.3	27.0	
Differentiation				0. 085
Well-moderate	148	52.6	NR	
Poor-undifferentiated	100	46.1	29.0	
Stage				0.003
I-II	173	54.4	74.0	
III -IV	75	36.4	24.0	
Vascular invasion				0.000
Absent	170	58.0	NR	
Present	78	30.5	20.0	
Relapse				0.000
Absent	148	58.5	NR	
Present	100	38.8	27	
Cytoplasmic ZEB2 expression				
Tumor tissues				0.026
Normal expression	98	41.8	27.0	
Overexpression	150	53.8	72.0	
Peritumoral liver tissues				0.001
Normal expression	130	42.5	28.0	
Overexpression	118	57.6	NR	
Nuclear ZEB2 expression				
Tumor tissues				0.549
Normal expression	230	49.9	38.0	
Overexpression	18	48.2	56.0	
Peritumoral liver tissues				0.280
Normal expression	191	48.5	38.0	
Overexpression	57	54.6	74.0	

* Mean age; NR, not reached; HbsAg, hepatitis B surface antigen; AFP, alpha-fetoprotein.

In peritumoral liver tissues, cytoplasmic overexpression of ZEB2 was negatively correlated with serum AFP level (*P* = 0.033, Table 1). Univariate analysis showed that patients with HCC who overexpressed ZEB2 also exhibited a longer survival time (mean survival time, 57.6 months) than patients with HCC who normally expressed ZEB2 (mean, 42.5 months; *P* = 0.001, log-rank test, Table 2, Figure 4B).

In combined analysis of cytoplasmic ZEB2 expression in HCCs and PLTs, the group with normal expression of ZEB2 in both HCC and PLT had the worst survival (mean survival time, 37.8 months), the group with normal expression of ZEB2 in either HCC or PLT showed moderate survival (mean survival time, 47.9 months), and the group with overexpression of ZEB2 in both HCC and PLT had the best survival (mean survival time, 59.5 months; *P* = 0.002; Figure 4C).

No correlation was found between nuclear expression of ZEB2 and clinicopathologic variables, such as patient's age, sex, AFP, cirrhosis, tumor size, tumor multiplicity, tumor differentiation, stage, vascular invasion and tumor relapse (*P*>0.05, Table 1). In univariate analysis, no significant association was demonstrated between patient survival and nuclear expression of ZEB2 neither in the tumor tissues nor in PLTs (*P*>0.05, Table 2).

### Independent prognostic factors of HCC

Since variables observed to have a prognostic influence on HCC patients by univariate analysis may covariate, ZEB2 expression and those clinicopathologic variables that were significant in univariate analysis (i.e., AFP levels, tumor size, tumor multiplicity, clinical stage, vascular invasion, and relapse) were further evaluated in multivariate analysis. Our result showed that overexpression of ZEB2 in peritumoral liver tissues was an independent prognostic factor for favorable patient overall survival (hazard ratio, 0.572; 95%CI, 0.374–0.873, *P* = 0.010; Table 3). Of the other variables, serum AFP level (*P*<0.0001), tumor multiplicity (*P* = 0.021) and vascular invasion (*P* = 0.008) were evaluated as well independent prognostic factors for patients' overall survival.

**Table 3:** Cox multivariate analyses of prognostic factors on overall survival.

Variables	Hazards ratio	95% CI	*P* value
AFP, ng/ml (≤20 *v* >20)	2.465	1.587–3.828	0.000
Tumor size, cm (≤5 *v* >5)	1.383	0.929–2.057	0.110
Tumor multiplicity (single *v* multiple)	1.594	1.073–2.368	0.021
Stage (I–II *v* III–IV)	1.245	0.760–2.038	0.385
Vascular invasion (absent *v* present)	1.915	1.184–3.098	0.008
Relapse (absent *v* present)	1.281	0.862–1.904	0.221
Cytoplasmic expression of ZEB2			
TT (normal *v* overexpression)	0.866	0.581–1.290	0.480
PLT (normal *v* overexpression)	0.572	0.374–0.873	0.010

CI, confidence interval; AFP, alpha-fetoprotein; TT, tumor tissue; PLT, peritumoral liver tissue.

### Correlations between expression of ZEB2 and E-cadherin or Vimentin in HCC

Additional IHC staining of E-cadherin and Vimentin has been performed on our HCC-TMA and thus, to analyze the potential correlation between expression of ZEB2 and E-cadherin or Vimentin in HCCs. Similarly, by utilizing the ROC curve analysis, the cutoffs for high expression of E-cadherin and Vimentin in HCC were defined when the cases with H score above the value of 160 and 75, respectively. High expression of E-cadherin and Vimentin were detected in 122/248 (49.2%) and 118/248 (47.6%) of HCCs, respectively (Figure S2). Further correlation analysis showed that there was no statistically significant correlation between cytoplasmic or nuclear expression of ZEB2 and E-cadherin or Vimentin in our HCC cohort (*P*>0.05, Fishers exact test).

## Discussion

ZEB2 has been suggested to mediate EMT and disease aggressiveness in various human cancers [14], [24]. Previous studies also showed increased levels of ZEB2 transcripts in association with invasion and metastasis in cancers with advanced stages [16], [25], [26]. However, the significance of ZEB2 expression in HCC and its effect on prognosis of HCC are still unclear. Thus, we performed the present large-scale study to investigate the expression dynamics of ZEB2 in tumor and peritumoral liver tissues and its clinicopathologic/prognostic significance in HCC patients using high-throughput TMA and IHC.

In this current study, the antibody used was a polyclonal anti-human ZEB2, which can detect an endogenous region of human ZEB2. Our result indicated that the staining of ZEB2 in HCCs predominantly displayed a cytoplasmic pattern, although nuclear staining was also observed in a few cases. This was consistent with the previous study, in which, Oztas [27] et al generated two anti-ZEB2 antibodies, clones 1C6 and 6E5, and assessed their immunoreactivity in a IHC study of cell lines and multiple tumor tissue arrays, yielded the similar results. These findings suggest that the observed expression pattern of ZEB2 by IHC was general to be a cytoplasmic pattern, while the nuclear localization of ZEB2 could be observed in only a few tumors. Interestingly, in our study, cytoplasmic expression pattern of ZEB2 was not only found in HCC tissues but also observed in peritumoral liver tissues. Similar findings were also documented in esophageal, gastric, colorectal and ovarian cancers [27], [28]. Moreover, the mean staining intensity of ZEB2 in HCCs was significantly lower than those in the peritumoral liver tissues. Consistent with our IHC findings, a recent report also indicated that strong cytoplasmic expression of ZEB2 could be detected in normal epithelial cells including hepatocytes, kidney tubules, stomach glandular and colon surface epithelium, and that ZEB2 appeared to be prevented from translocating into nucleus in these tissues [27]. In addition, cytoplasmic overexpression of ZEB2 in HCC tissues was found to correlate with low serum AFP level, small tumor size and well tumor differentiation, and negative correlation of overexpression of cytoplasmic ZEB2 in PLTs and serum AFP level was also observed. These data suggested that the up-regulated expression of ZEB2 in tumor and peritumoral tissues of HCC might be a protective role for ZEB2 against tumorigenesis. However, the underlying mechanisms still need further to be clarified.

The most important finding of the present study was the prognostic significance of cytoplasmic ZEB2 expression in HCC and peritumoral liver tissues. In this study, overexpression of cytoplasmic ZEB2 was associated closely with HCC patient longer survival time as evidenced by univariate analysis in both tumor and peritumoral liver tissues. Strikingly, HCC patients with ZEB2 overexpression in both tumor and peritumoral tissues have the longest survival time among the subgroups of different ZEB2 expression status. Moreover, no significant association of ZEB2 expression and E-cadherin or Vimentin expression was found in our HCC cohort. With regards to the role of ZEB2 in the progression of human cancers, some of the results are totally contradictory. On one hand, it has been reported that elevated ZEB2 expression correlated positively with adverse patient survival in breast, ovarian, kidney, oral and bladder cancers [14], [15], [16], [17]. Also, ZEB2-mediated upregulation of EMT and tumor invasion related genes, such as E-cadherin, vimentin and metalloproteases, have been indicated [16]. On the other hand, ZEB2 was shown to inhibit expression of cyclin D1 and be partly responsible for hTERT repression [13], [29]. In addition, downregulation of ZEB2 in HCC cell lines and cancers was found to be mediated by aberrant promoter methylation [30]. Considering that ZEB2 takes part in the TGF-β pathway and the effects of TGF-β on cell are variable and depend on various factors including cell type and physiological state of tissues [31], [32], it is not very difficult for us to understand that the function of ZEB2 and its underling mechanism(s) to impact cancer progression may be tumor-type specific. These data, collectively, might also provide a possible explanation that in our present study, why expression of ZEB2 was not significantly correlated with E-cadherin and Vimentin in our HCC tissues.

In our study, cytoplasmic ZEB2 overexpression in peritumoral liver tissues was further evaluated as an independent factor of favorable survival for HCC patients. We also found that cytoplasmic expression in the peritumoral liver tissue is better able to stratify cancer specific survival than expression in the tumor tissue. To our best knowledge, few studies that focus on peritumoral liver tissues on patient survival have been indicated. Zhu et al [5] found that high expression of macrophage colony-stimulating factor in peritumoral liver tissues was correlated with poor survival after curative resection of HCC. Ezaki et al [33] showed that higher peritumoral expression of thymidine phosphorylase was associated with a higher incidence of postoperative recurrence in HCC. Also, Okamoto et al [34] reported that a specific gene profile in peritumoral liver tissue could predict multicentric occurrence or recurrence of HCC. In accordance with previous study [13], our observations suggest that ZEB2 may act as a tumor suppressor gene in HCC. Furthermore, we found that ZEB2 overexpression was more frequently seen in peritumoral hepatocytes compared to HCC cells, which might provide a more powerfully protective role to inhibit HCC growth. It might provide a possible explanation for why ZEB2 expression in the adjacent tissue better prognosticates than the expression in the tumor tissue. Together with these data, the present study implies that postoperative adjuvant therapies should target not only the residual cancer cells, but also the soil to make it resistant to tumor growth. Anyway, since majority of the previous studies was focus on the expression status of ZEB2 in tumor tissues of different human cancers, our data suggested, for the first time, a potential role of ZEB2 expression in peritumoral liver tissues in tumor development of HCC.

In summary, our results provide interesting and new information that overexpression of cytoplasmic ZEB2 in HCC and peritumoral liver tissues may be important in the acquisition of a favorable phenotype, suggesting that overexpression of cytoplasmic ZEB2 by peritumoral liver tissues, as detected by IHC, may predict the postoperative survival of patients with HCC, and it might be a helpful criterion to optimize individual therapeutic management.

## Supporting Information

Figure S1 **The expression of ZEB2 in HCC and adjacent liver tissues by Western blotting.** Down-regulated expression of ZEB2 was detected in 9/12 cases of HCC tissues compared to adjacent liver tissues. *T*, HCC tissue; *N*, peritumoral liver tissue.
(TIF)

Figure S2 **The expression patterns of E-cadherin and Vimentin in HCC tissues by immunohistochemistry.** Overexpression of E-cadherin and Vimentin were shown in representative cases of patient with HCC.
(TIF)
