# Supplementary figures and images for: Overexpression of ZEB2 in Peritumoral Liver Tissue Correlates with Favorable Survival after Curative Resection of Hepatocellular Carcinoma
Source: PLoS One. 2012 Feb 29;7(2):e32838. doi: 10.1371/journal.pone.0032838 (PMC3290607; doi:10.1371/journal.pone.0032838)

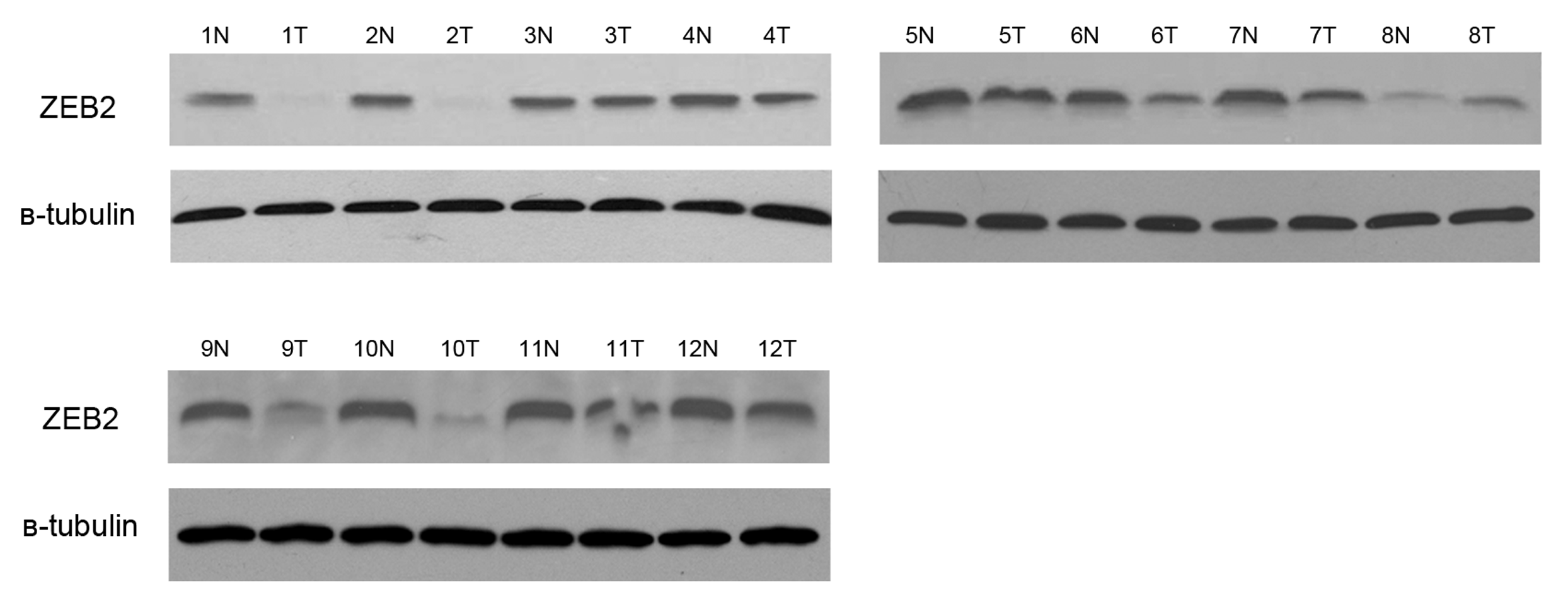

Supplement: Figure S1 — The expression of ZEB2 in HCC and adjacent liver tissues by Western blotting. Down-regulated expression of ZEB2 was detected in 9/12 cases of HCC tissues compared to adjacent liver tissues. T, HCC tissue; N, peritumoral liver tissue. (TIF) [file pone.0032838.s001.tif]

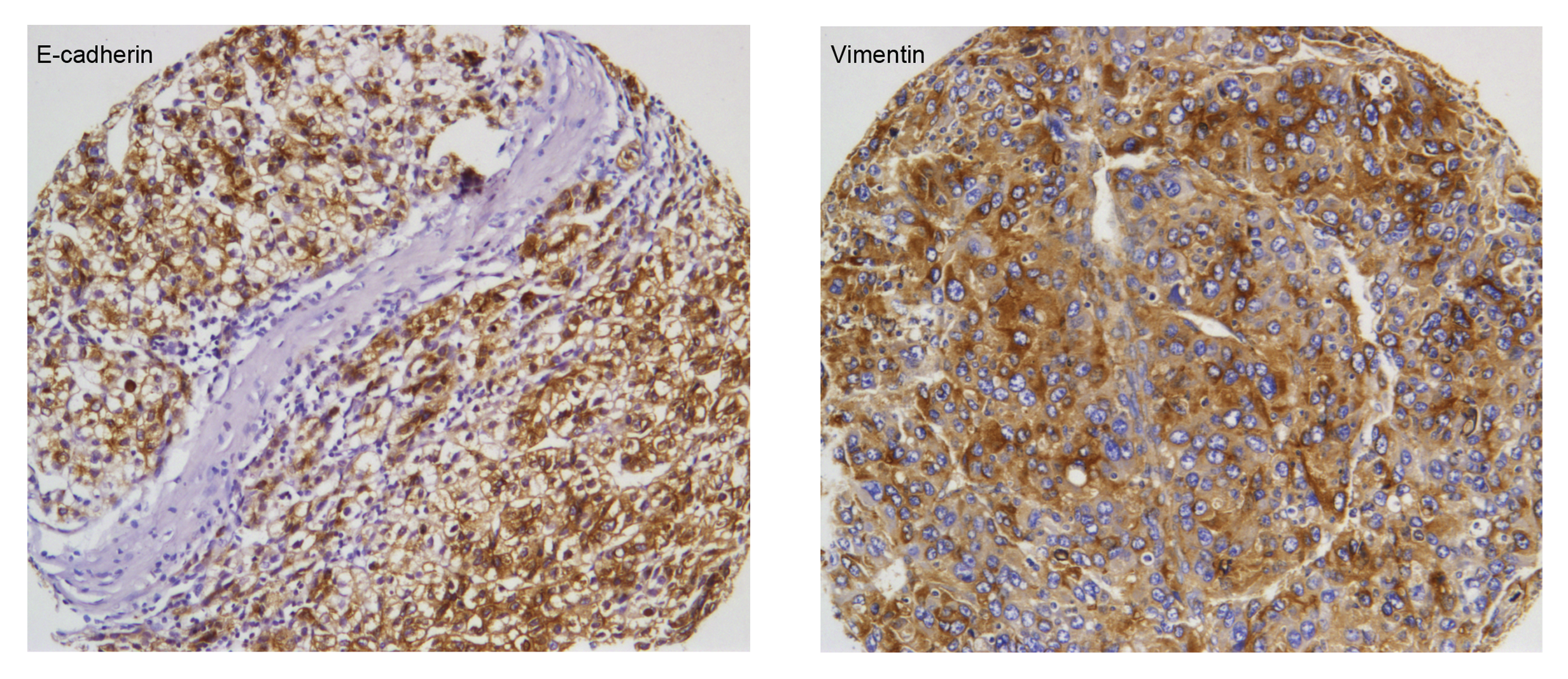

Supplement: Figure S2 — The expression patterns of E-cadherin and Vimentin in HCC tissues by immunohistochemistry. Overexpression of E-cadherin and Vimentin were shown in representative cases of patient with HCC. (TIF) [file pone.0032838.s002.tif]
